# Economic evaluation of third-line neratinib plus capecitabine versus lapatinib plus capecitabine with HER2+ metastatic breast cancer

**DOI:** 10.3389/fonc.2023.1221969

**Published:** 2023-08-09

**Authors:** Lanqi Ren, Ning Ren, Yu Zheng, Yibei Yang, Qiaoping Xu

**Affiliations:** ^1^ Fourth Clinical Medical College of Zhejiang Chinese Medical University, Hangzhou, Zhejiang, China; ^2^ Second Clinical Medical College of Zhejiang Chinese Medical University, Hangzhou, Zhejiang, China; ^3^ Department of Clinical Pharmacology, Key Laboratory of Clinical Cancer Pharmacology and Toxicology Research of Zhejiang Province, Affiliated Hangzhou First People’s Hospital, Cancer Center, Zhejiang University School of Medicine, Hangzhou, China

**Keywords:** breast cancer, chemotherapy, pharmaceutical economics, Markov model, cost-effectiveness analysis

## Abstract

**Background:**

Breast cancer (BC) is one of the most common malignant tumors in women. In addition, human epidermal growth factor receptor 2-positive (HER2+) BC is overexpressed in 25% of BC patients, resulting in the predicament of poor prognosis. Although first- and second-line treatments have been established, optimum third-line treatment is still mired in controversies for HER2+ metastatic BC (mBC). Therefore, this study analyzes the cost-effectiveness of neratinib plus capecitabine (N+C) and lapatinib plus capecitabine (L+C) over a 5-year time horizon from a payer perspective.

**Methods:**

A half-cycle corrected four-state Markov model was established to simulate the course of BC events and deaths in N+C and L+C armed patients. The data of this model were derived from NCT01808573 trail and other published literatures. One-way deterministic sensitivity analysis (DSA) was conducted to investigate the impact of variables and probabilistic sensitivity analysis (PSA) was performed based on second-order Monte Carlo simulation. In addition, subgroup analysis was performed to verify its cost-effectiveness in China.

**Result:**

The base-case results found that N+C was in dominant position in 82.70% of the generation scenarios, providing an improvement of 0.17 quality-adjusted life-years (QALYs) and a reduction of $1,861.28 compared with L+C. The ICER was $-1,3294.86/QALY, which did not exceed the willingness to pay (WTP) threshold, while in subgroup, the ICER decreased to $-2,448.17/QALY.

**Conclusion:**

This analysis indicated that the combination of neratinib plus capecitabine is likely to be cost-effective in comparison with lapatinib plus capecitabine in patients with HER2+ mBC who continues to progress during or after second-line HER2-targeted therapy. So neratinib plus capecitabine can become a third-line treatment option.

## 1 Introduction

Breast cancer (BC) is one of the most common cancers in women, and its incidence continues to increase worldwide (1). Human epidermal growth factor receptor 2-positive (HER2+) BC, which accounts for approximately 25% of all BC, has a higher malignancy rate and a poorer prognosis than HER2− BC (2). HER2 is a transmembrane receptor tyrosine kinase in the epidermal growth factor receptor family, which causes tumor recurrence and central nervous system metastasis (3, 4). In the past three decades, astonishing strides have been made in the treatment of patients with HER2+ metastatic BC (mBC). Several effective anti-HER2–targeted agents have significantly improved their prognosis, including monoclonal antibodies (mAbs), tyrosine kinase inhibitors (TKIs), and antibody-drug conjugates (ADCs) (5). The mAbs pertuzumab, trastuzumab, and margetuximab were approved by US Food and Drug Administration (FDA) in 1998, 2012, and 2020, respectively (6). Lapatinib was the first-generation TKI approved in 2007, after which neratinib and tucatinib were approved in 2020, showing an improvement in combination with capecitabine (7). Trastuzumab emtansine (T-DM1) and trastuzumab deruxtecan (T-DXd; DS-8201) were the ADCs approved by the FDA for the treatment of HER2+ mBC in 2013 and 2022 (5).

As first-line treatment, dual HER2-targeted mAbs, pertuzumab + trastuzumab, in combination with a taxane has a favorable therapeutic effect in a large proportion of patients (7). In second-line treatment, nowadays, T-DXd is found to be the preferred option compared with T-DM1 (8, 9). However, there is still no standard protocol for patients with HER2+ mBC who continue to progress during or after second-line HER2-targeted therapy. On the one hand, the penetration of anti-HER2–targeted agents in the blood–brain barrier is thought to be limited. On the other hand, approximately 20% of patients eventually die due to primary or acquired treatment resistance (10). Since there is no standard treatment for patients with trastuzumab, pertuzumab, and T-DXd resistance (4), the treatment of patients with drug resistance is still a great challenge. Therefore, research has focused on strategies to overcome resistance to HER2+ mBC therapies.

Recently, a large number of clinical trials concentrating on third-line treatment have come to conclusion that small-molecule TKIs have a tremendous effect in improving the prognosis of HER2+ mBC, rendering the advantages of oral administration, multi-target therapy, and low toxicity (11–14). Lapatinib is a reversible TKI that has intracranial activity and increases the survival of HER2+ BC patients with brain metastases, according to Khan et al., in a meta-analysis (15). Lapatinib plus capecitabine has a tangible increase in progression-free survival (PFS) but no alteration in overall survival (OS) (16). Unlike lapatinib, neratinib is an irreversible pan-HER inhibitor with greater effect and greater toxicity than lapatinib. In the exteNET clinical trial (17), it showed a high-response rate of 73% as well as a high toxicity such as diarrhea, the most serious adverse event (AE). However, diarrhea often occurs within the first month after treatment, so antidiarrheal prophylaxis can be performed early in treatment, according to NCT00878709 trial (18). Since 2020, neratinib has been administered in combination with capecitabine in patients who have received at least two prior anti-HER2 therapies (19, 20), which seemingly illuminates the brand-new future for HER2+ mBC treatment.

However, new treatments may be associated with higher costs but minimal improvements in treatment outcomes. In order to address the question, we compare the cost-effectiveness and safety of lapatinib plus capecitabine and neratinib plus capecitabine in patients with HER2+ mBC.

## 2 Methods

### 2.1 Model design

**Figure 1:**
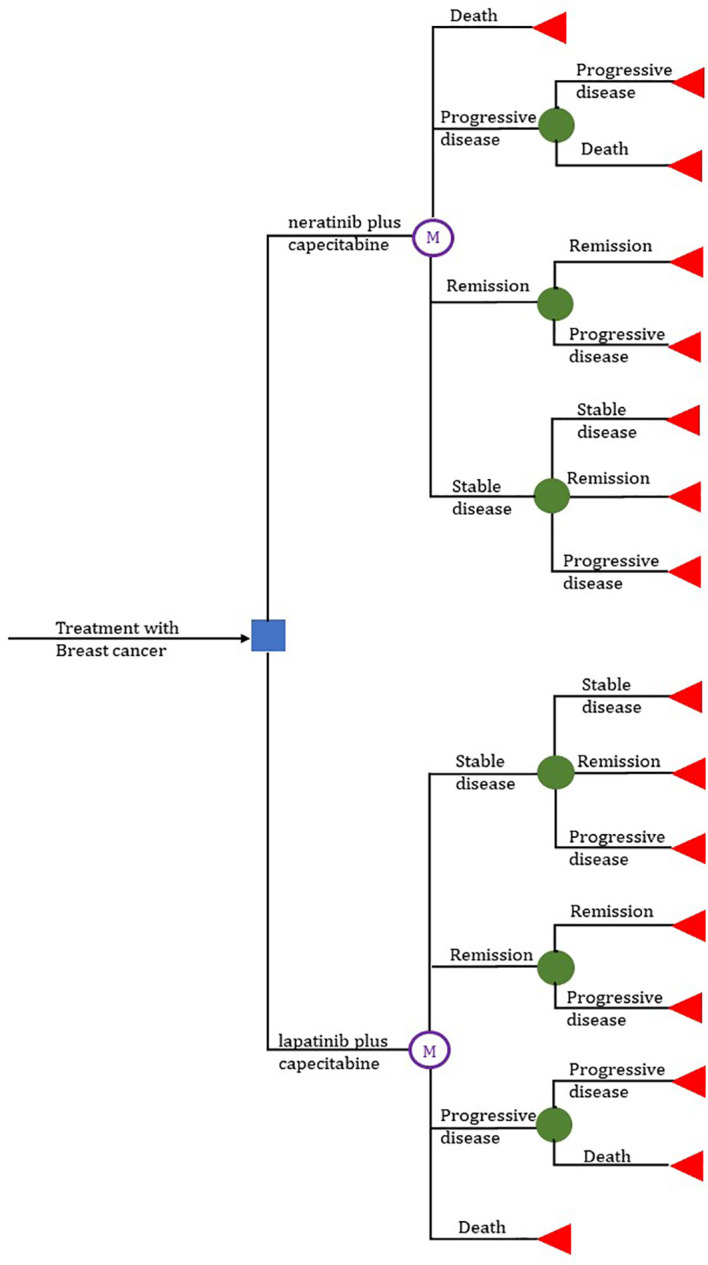
Treeage and Markov model.

We used TreeAge Pro Suite 2022 (TreeAge Software, Williamstown, MA, USA) to construct decision tree and Markov model. We assumed a patient population based on the NCT01808573 trial (21); eligible patients were included in our model and randomly assigned to receive N+C or L+C, and then cost-effectiveness was analyzed in terms of OS, PFS, duration of response (DoR), and relative risk (RR). Patients were in various health states basing the NCT01808573 trial before treatment. We chose 5 years as a suitable time horizon, during which they moved into four health states including stable disease (SD), progressive disease (PD), remission (RE), until entering the absorptive state death (DE) (**Figure 1**). The cycle length was set to 21 days to correspond to the chemotherapy cycle length.

Our measured outcome parameters were total costs, quality-adjusted life-years (QALYs), incremental cost-effectiveness ratios (ICERs) and net monetary benefit (NMB). Among them, cost and survival were estimated using a semi-cyclical correlation and an annual discount rate of 3% (22). According to the recommendation of Chinese guidelines for pharmacoeconomic evaluations, if the ICER is lower than *per capita* GDP, the strategy is cost-effective; if the ICER is between *per capita* GDP and triple *per capita* GDP, the strategy is acceptable; and if the ICER was greater than triple *per capita* GDP, the strategy is not worthwhile (23). China got a per capita GDP of $12,000 in 2022 (24). Therefore, we set triple *per capita* GDP as willingness to pay (WTP) threshold, which was $36,000/QALY.

### 2.2 Study population

There were 621 participants in the NCT01808573 trial, but only 493 were treated and followed for outcomes, which resulted in a total of 493 patients in the simulation: 241 in the N+C group and 252 in the L+C group. Group N+C received neratinib (240 mg, once daily, orally with food, continuously on 21-day cycles) and capecitabine (1500 mg/m^2^, twice a day, orally administered with water within 30 min after meals, on days 1–14 of 21-day cycles). In addition, loperamide was administered in the N+C group with the first dose of neratinib (initial dose, 4 mg) in cycle 1, followed by 2 mg every 4h for the first 3 days. Thereafter, loperamide 2 mg was administered every 6h–8h until the end of the first cycle, regardless of whether the patient developed diarrhea. The L+C group received lapatinib (1250 mg, once daily, orally, continuously on 21-day cycles) and capecitabine (2000 mg/m^2^, twice a day, orally administered with water within 30 min after meals, on days 1–14 of 21-day cycles).

### 2.3 Transition probabilities data

The transition probability of a Markov state transition model in pharmacoeconomic evaluation refers to the likelihood that a patient will transition from one state to another within a cycle. In this study, the DEALE method was used to transform the length of time into a rate index, and then the rate index into a probability (25, 26). The clinical efficacy indicators based on which the probability of metastasis was calculated were mainly RR, OS, PFS, and DoR. The relevant data were derived from clinical trials, and the transition probability calculation procedure was shown in **Table 1**.

**Table 1:** The key parameters of the metastatic breast cancer model with N+C and L+C.

Variable/probability	Formula	Best estimate	SA range	Distribution
––N+C				
RR		0.63		
OS		24		
PFS		8.8		
DoR		8.5		
Stable→Stable (Nss)	1-Nsp-Nsr	0.575	0.5175–0.6325	Beta
Stable→Remission(Nsr)	1-exp(-RR/3)	0.189	0.1701–0.2079	Beta
Stable→Relapse(Nsp)	Nrp*4	0.236	0.2124–0.2596	Beta
Remission→Remission(Nrr)	1-Nrp	0.941	0.8469–1.0000	Beta
Remission→Relapse(Nrp)	1-exp[-0.75*In(2)/(DoR)]	0.059	0.0531–0.0649	Beta
Relapse→Relapse(Npp)	1-Npd	0.966	0.8694–1.0000	Beta
Relapse→Death(Npd)	1-exp[-0.75*In(2)/(OS-PFS)]	0.034	0.0306–0.0374	Beta
––L+C				
RR		0.7		
OS		22		
PFS		6.6		
DoR		5.6		
Stable→Stable (Lss)	1-Lsp-Lsr	0.44	0.3960–0.4840	Beta
Stable→Remission(Lsr)	1-exp(-RR/3)	0.208	0.1872–0.2288	Beta
Stable→Relapse(Lsp)	Lrp*4	0.352	0.3168–0.3872	Beta
Remission→Remission(Lrr)	1-Lrp	0.912	0.8208–1.0000	Beta
Remission→Relapse(Lrp)	1-exp[−0.75*In(2)/DoR)]	0.088	0.0792–0.0968	Beta
Relapse→Relapse(Lpp)	1-Lpd	0.967	0.8703–1.0000	Beta
Relapse→Death(Lpd)	1-exp(−0.75*In(2)/(OS-PFS)]	0.033	0.0297–0.0363	Beta
Discount rate for costs and QALYs		3% per year		
Health state utilities				
No recurrence (chemotherapeutic period)		0.74	0.0592–0.888	Beta
No recurrence (after chemotherapy)		0.94	0.752–1.000	Beta
local recurrence (in the first year)		0.74	0.592–0.888	Beta
Remission		0.85	0.680–1.000	Beta
Relapse		0.5	0.400–0.600	Beta

RR, relative risk, (OS−PFS)/OS; OS, overall survival; PFS, progression-free survival; DoR, duration of response; QALY, quality-adjusted life-year.

### 2.4 Utility data

Health utility value is the weight of a health state relative to full health, an indicator to evaluate the degree of satisfaction with a health state, and a comprehensive index to reflect the health status of an individual. The value ranges from 0 to 1, with 0 representing death and 1 representing full health. Health utility values used to calculate QALYs were derived from published literature (27), and were shown in **Table 1**.

### 2.5 Cost data

Costs considered included hospitalization, health examination, concomitant medications, management of serious AEs, and chemotherapy drugs. Patients in the state of PD were assumed to pay for the cost of hospitalization and concomitant medications while patients in the state of SD and RE were supposed to have a regular health examination involving CT scan, renal panel, liver function test, and so on. Based on clinical data, we included grades 3 and 4 AEs that occurred in more than 3% of patients and differed significantly between treatments. To calculate the drug dose of the drug, a typical patient was assumed to have a surface area of 1.67m^2^. Chemotherapy drug prices were obtained from the lowest price in the Pharmstore (28), other drug prices were obtained from the relevant published literature (27, 29–31) or local hospital, and they were expanded to 2022 based on an online consumer price index (CPI) calculator. Cost-related data were shown in **Table 2**.

**Table 2:** Model input for costs.

Cost ($2,022)	N+C	L+C	SA range	Distribution
Cost of chemotherapeutics				
Neratinib (per 40 mg)	13.46		12.114–14.806	Gamma
Lapatinib (per 250 mg)	16.12		14.508–17.732	Gamma
Capecitabine (per 150 mg)	0.6		0.540–0.660	Gamma
Total	10176.6	11541.6		
Cost of AEs				
Loperamide (per 2 mg)	0.23		0.207–0.253	Gamma
Diarrhea (per event)	4083.63		3675.267–4491.993	Gamma
PPE syndrome (per event)	2316		2084.400–2547.600	Gamma
Vomiting (per event)	945.63		851.067–1040.193	Gamma
Fatigue (per event)	1158.04		1042.236–1273.844	Gamma
Anemia (per event)	7350.98		6615.882–8086.078	Gamma
Total	16183.38	15854.28		
Cost for others				
Hospitalization	3200		2880.000–3520.000	Gamma
Concomitant medications	2176		1958.400–2393.600	Gamma
Health examinations	4552		4096.800–5007.200	Gamma
Total	9928	9928		

AEs, advanced events. PPE, Palmar-Plantar Erythrodysesthesia.

### 2.6 Sensitivity analysis

To explore the uncertainty in the model, we performed one-way deterministic sensitivity analysis (DSA) and probabilistic sensitivity analysis (PSA). In PSA, we determined the most suitable distribution of the parameters according to their types, using the beta distribution to represent the uncertainty of utility, probability and proportion, and the gamma distribution to represent the uncertainty of cost. Then, Monte Carlo simulations were used to randomly extract key model parameters and repeated 1,000 times to obtain the hypothetical cohort. Based on the results of PSA, a cost-effectiveness curve was drawn to visually show the ratio of cost to effectiveness of the two schemes under the premise that WTP was met. In addition, we relied only on the value analysis of these expected ICERs, sometimes with negative QALYs and negative costs, making the mean and median ICERs difficult to interpret.

To investigate the effect of each parameter on the ICERs, we also performed one-way DSA with all uncertain parameters, such as transition probability and discount rate, varied within ±10% or 95% confidence intervals (95% CI) of their baseline values. One-way DSA results were presented as tornado diagram.

Finally, to analyze the situation of negative ICERs better, we performed NMB analysis and generated a cost-effectiveness acceptability curve using these NMB values instead of ICERs, since NMB was more effective when in the face of negative ICERs (27). A cost-effectiveness threshold of $36,000/QALY was set for the NMB analysis.

### 2.7 Subgroup analysis

Cost-effectiveness analysis was performed in subgroups of Asians in the trial to investigate the applicability of the results to Asians. In a subgroup analysis of N+C versus L+C (32), median PFS (7.0 vs. 5.4 months, *P* = 0.0011) and median OS (23.8 months vs. 18.7 months, *P* = 0.185) were obtained. In addition, the DoR of N+C was 11.1 months, and that of L+C was 4.2 months (*P* < 0.0001).

## 3 Results

### 3.1 Base-case analysis

**Figure 2:**
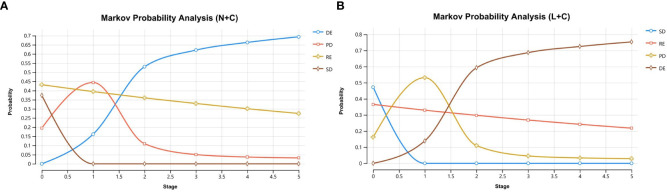
Markov cohort analysis **(A)** N+C cohort **(B)** L+C cohort. These curves show the output of the N+C and L+C models. The horizontal axis shows time (years) and the vertical axis shows the proportion of people.

Cumulative lifetime costs, QALYs, incremental QALYs, incremental costs, ICERs, mortality rates, and NMB were calculated. The proportions of SD (0%), RE (27.4%), PD (3.2%), and DE (69.3%) in the N+C were calculated by the Markov model (**Figure 2A**). In addition, the proportions of the four distributions of the L+C were SD (0%), RE (21.8%), PD (2.8%), and DE (75.3%) (**Figure 2B**).

**Figure 3:**
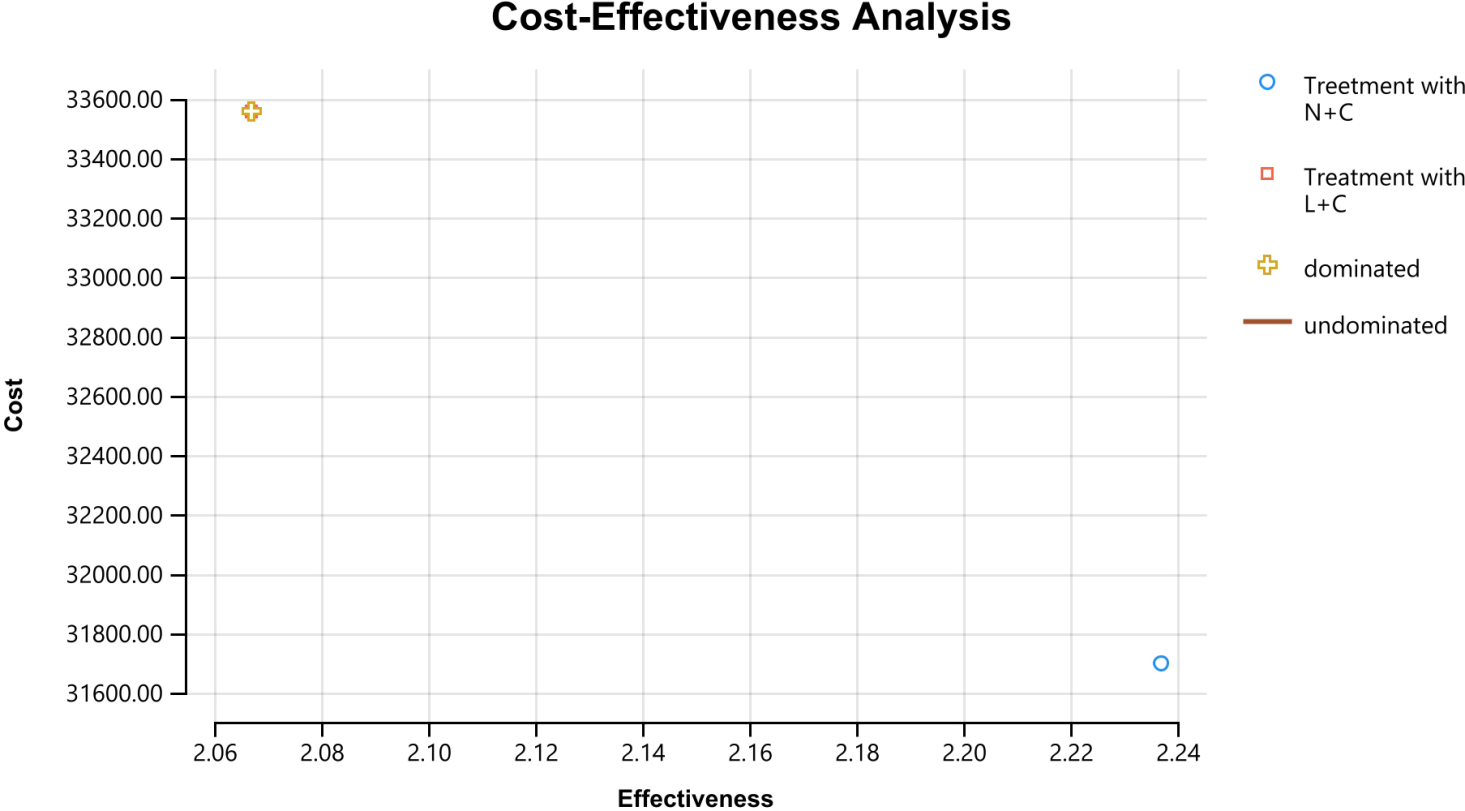
Results of a cost-effectiveness analysis of breast cancer patients. Cumulative lifetime costs are shown on the vertical axis, and QALYs gained are shown on the horizontal axis.

Clearly, under our baseline assumptions, the model results showed that N+C was the dominant strategy: It not only cost less but also had more QALYs compared with the L+C. The average price of the N+C was $1,861.28 lower than that of the L+C. Treatment with neratinib, as compared with the lapatinib group, was estimated to result in an incremental 0.17 QALYs (2.24 QALYs vs. 2.07 QALYs). Applying the incremental analysis principle, the ICER of $13,294.86/QALY was obtained for the N+C in comparison with the L+C (**Table 3** and **Figure 3**).

**Table 3:** Cost and effect of N+C and L+C within 5 years.

Item	status	N+C	L+C	Deviation
Effect	PFS/%	27.4	21.8	5.6
	DE/%	69.3	75.3	−6
	QALY/Year	2.24	2.07	0.17
Effect(subgroup)	PFS/%	29.9	32.3	−2.4
	DE/%	65.2	66.4	−1.2
	QALY/Year	2.49	2.26	0.23
Cost		31803.33	33664.61	−1861.28
Cost(subgroup)		30742.90	31305.98	−563.08
ICER				−13294.86
ICER(subgroup)				−2448.17
NMB		39788.07	32876.84	
NMB(subgroup)		49022.57	41265.83	

ICER, incremental cost-effectiveness ratio; QALY, quality-adjusted life-year; NMB, net monetary benefit; PFS, progression-free survival; DE, death.

### 3.2 General safety

The most common grades 3 and 4 AEs in both groups were diarrhea, Palmar-Plantar Erythrodysesthesia (PPE) syndrome, vomiting, fatigue, and anemia. Grades 3 and 4 diarrhea were recorded in 74 patients (30.7%) in the N+C and 39 (14.3%) in the L+C, which was the most common AEs in both groups. PPE syndrome was the most common AEs of capecitabine, and the incidence was also high with 29 cases (12.0%) in N+C and 35 cases (13.8%) in L+C. The incidence of AEs was shown in the **Table 4**.

**Table 4:** Adverse events (G3 and G4) for N+C versus L+C.

AEs	N+C	L+C
Diarrhea	74(30.7%)	39(14.3%)
PPE syndrome	29(12.0%)	35(13.8%)
Vomiting	12(5.0%)	6(2.4%)
Fatigue	9(3.7%)	10(4.0%)
Anemia	6(2.4%)	11(4.4%)

AEs, advanced events. PPE, Palmar-Plantar Erythrodysesthesia.

### 3.3 Sensitivity analysis

**Figure 4:**
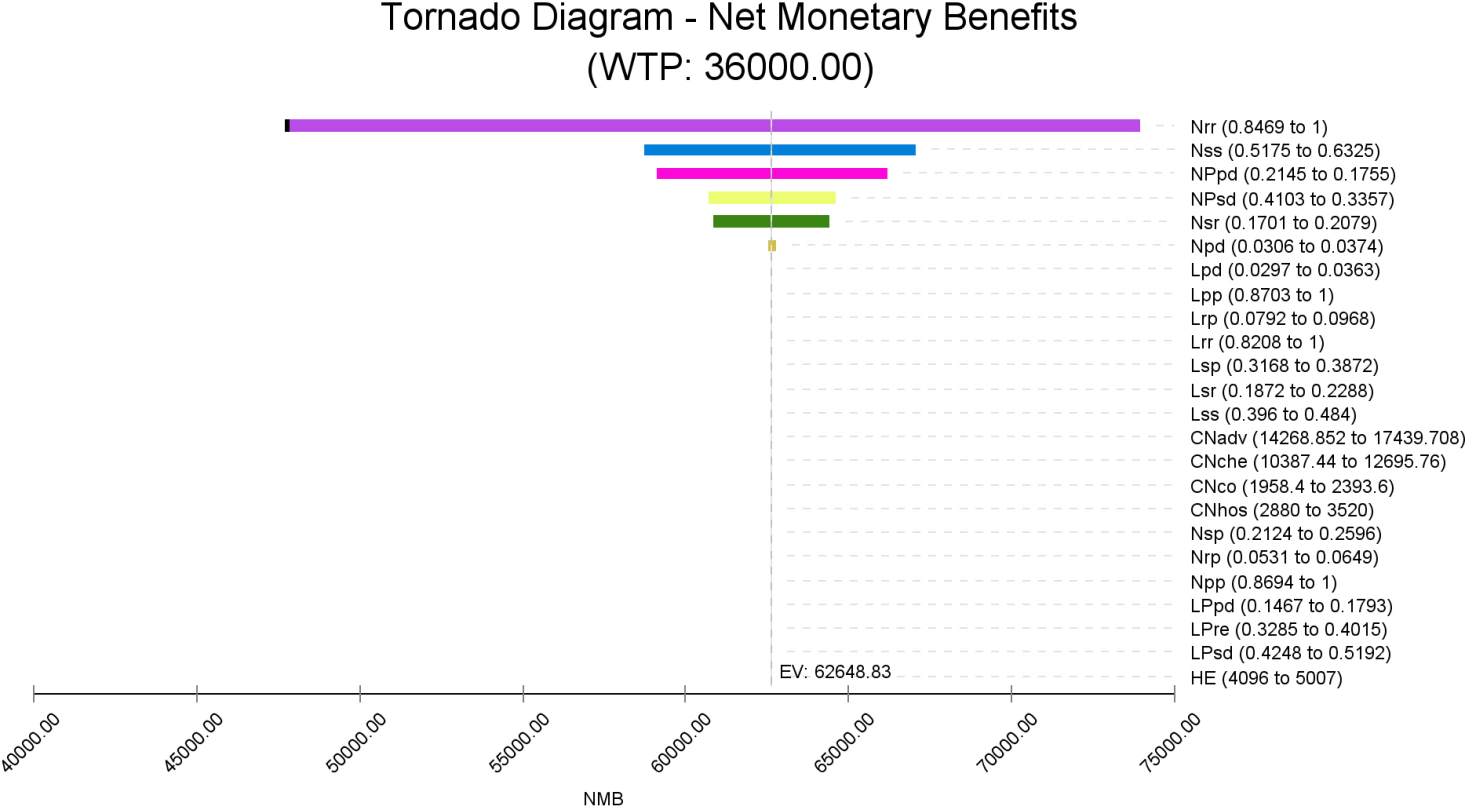
Tornado diagram. The tornado diagram represents the cost per unit QALY gained in a one-way sensitivity analysis of the N+C versus L+C strategy. The widths of the bars indicate the range of the results when the variable changes within the sensitivity analysis range. The vertical dashed line represents the results for the base case.

One-way DSA showed that the five most influential parameters were the transition probability of N+C from remission to remission (Nrr), the transition probability of N+C from stable to stable (Nss), the probability of N+C in the initial state of progressive phase (NPpd), the probability of N+C in the initial state of stable phase (NPsd), and the transition probability of N+C from stable to remission (Nsr). When the threshold was set at $36,000/QALY, only Nrr had a certain influence on the ICER results in the sensitivity analysis range. In addition, although the health utility values in this study were derived from only one article, we found that it had little influence on the results (**Figure 4**).

**Figure 5:**
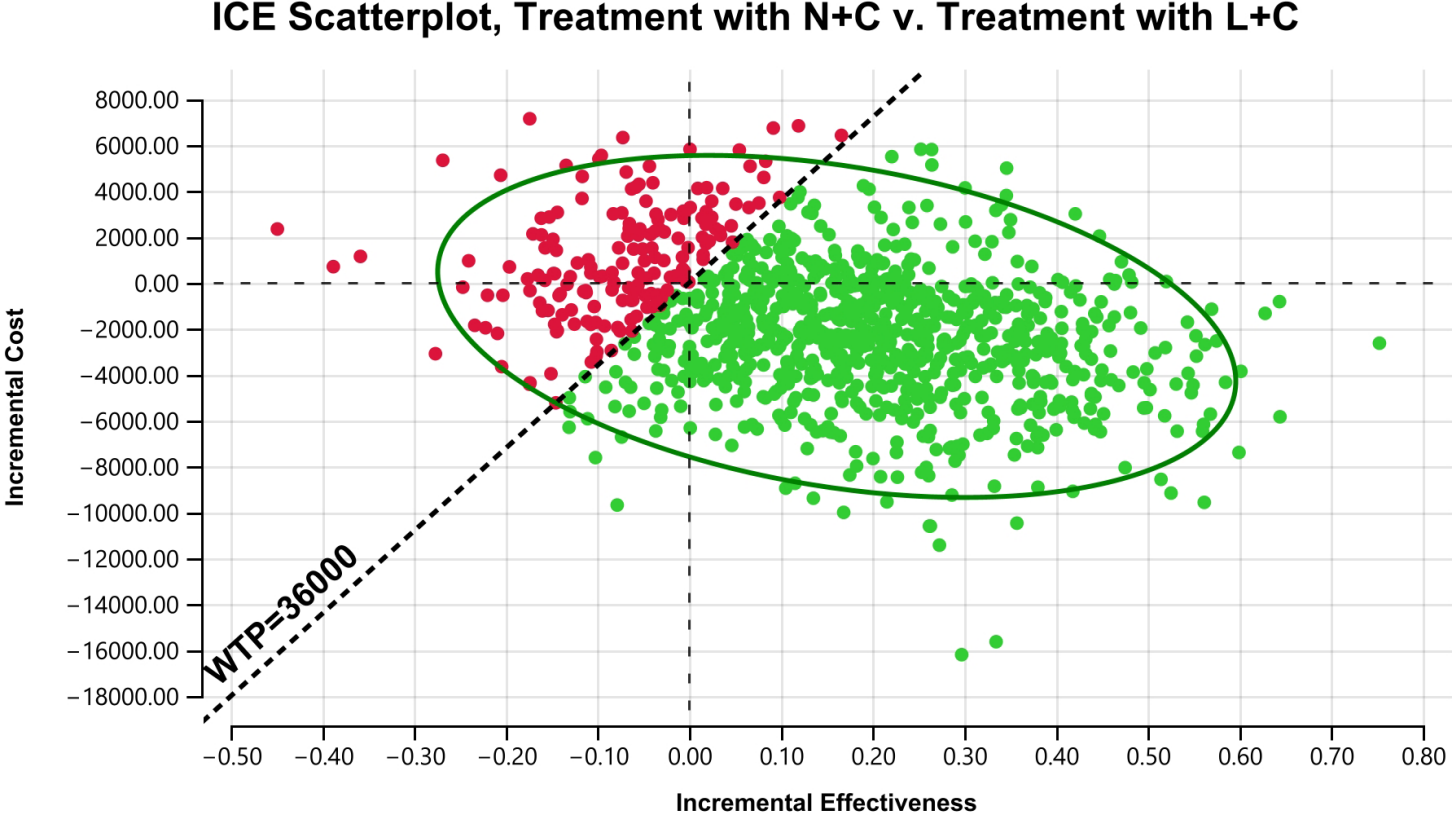
Probabilistic outcome of the incremental cost-benefit difference between N+C and L+C treatment in a simulated cohort. The vertical axis represents incremental cost and the horizontal axis represents QALYs obtained. The slash line represents the upper limit of willingness to pay, and data points below the slash line are cost-effective.

When it came to PSA, scatter plots were obtained based on the Monte Carlo simulation results. The horizontal and vertical axes represented the incremental effectiveness and incremental cost of the N+C versus L+C, and each scatter represented the ICERs for the N+C versus L+C in patients with HER2+ mBC. According to the figure (**Figure 5**), 82.70% of the generation scenarios (represented by green dots) favored the N+C scheme, among which 64.5% of the cases were endowed with absolute effectiveness because of the negative ICERs, 13% of the cases have higher cost and better effect with the ICERs not exceeding the WTP threshold, and 5.2% of the cases still have relative advantages compared with L+C although the ICERs exceeded the WTP threshold. However, in the other 17.30% of the generation scenarios (represented by red dots), L+C was more likely to be the dominant solution. In addition, scatter points were more concentrated in the ICERs range (ellipse), demonstrating that the ICERs analysis results of these two schemes were relatively robust.

**Figure 6:**
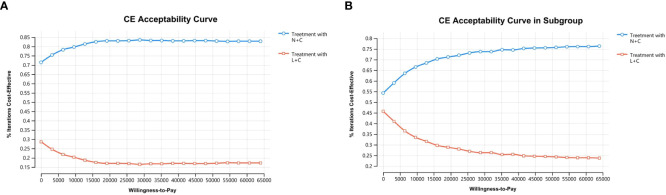
Cost-effectiveness acceptability curve: **(A)** the whole group and **(B)** the subgroup. The curves show the probability of the net gain for each strategy for different WTP thresholds in the N+C and L+C cohorts. The vertical axis represents the probability of cost-benefit generation. The horizontal axis represents the WTP threshold for obtaining an additional QALY.

In NMB analysis, a cost-effectiveness acceptability curve can be derived for a cohort of 1,000 patients with HER+ mBC. Within the WTP range (0–64000), the probability of cost-effectiveness of L+C gradually decreased and became stable when WTP reached $20,000/QALY. In contrast, the probability of cost-effectiveness of N+C gradually rose and reached a plateau when WTP reached $20,000/QALY. However, no matter how the WTP changes, the acceptance probability of N+C was always much higher than that of L+C within the scope of our analysis, indicating that the N+C had a higher acceptance probability and was the preferred strategy (**Figure 6A**).

### 3.4 Subgroup analysis

In the Asian subgroup analysis, the cumulative costs and effectiveness were $30,742.90 and 2.29 QALYs for the N+C cohort, and $31,305.98 and 2.26 QALYs for the L+C cohort, resulting in the ICER of $-2,448.17/QALY (**Table 3**). PSA showed that within the threshold of $36,000/QALY, N+C was still the dominant strategy, although the probability of N+C being accepted was lower than that of the whole group analysis (**Figure 6B**).

## 4 Discussion

In this study, we aimed to determine the long-term cost and long-term effectiveness of two treatment strategies for third-line treatment of HER2+ mBC. Therefore, a 5-year Markov model was run to summarize the long-term costs and QALYs of the two treatment strategies in the NCT01808573 trial. Our study showed that the N+C was less costly ($31,803.33 vs. $33,664.61) and more effective (2.24 QALYs vs. 2.07 QALYs) with a greater probability of producing a favorable NMB in contrast with L+C ($39,788.07 vs. $32,876.84), indicating that the N+C was superior to the L+C. Also, the cost-effectiveness of N+C versus L+C remained robust in PSA. Of the hypothetical 1,000 scenarios generated in Monte Carlo simulations, 82.7% indicated that N+C was cost-effective compared with L+C. In one-way DSA, the most significant effect was Nrr, which was explained by the small difference in health outcomes predicted by our model between the two groups, as the N+C cohort had only 0.17 more QALYs than L+C. Moreover, it is noteworthy that our subgroup analysis further showed the applicability of the N+C in Asia.

The finding that N+C was cost-effective compared with L+C was not astonishing. Many clinical trials and analyses have demonstrated the effectiveness of neratinib, which has been shown to be effective in both the prevention and treatment of HER2+ mBC. The CNS subgroup analysis of the NCT01808573 trial by Hurvitz et al. found a mean PFS of 7.8 months in the N+C group and 5.5 months in the L+C group at 24 months, and a mean OS of 16.4 months and 15.4 months at 48 months, respectively. These analyses demonstrated improved outcomes with N+C compared with L+C in patients with HER2+ MBC CNS metastases (33). In the NCT00915018 trial, neratinib-paclitaxel delayed the onset and decreased the frequency of central nervous system progression, although there was no clear advantage over trastuzumab with respect to PFS in first-line HER2+ mBC (12). The TBNRC022 trial showed that neratinib plus capecitabine was active in brain metastases from refractory HER2+ BC and that chemotherapy enhanced the efficacy of HER2-directed therapy in the brain. Neratinib was associated with longer median PFS (5.5 vs. 3.1 months) and longer median OS (13.3 vs. 15.1 months) than lapatinib (34). Martin et al. in a prospective subgroup analysis showed a greater benefit of neratinib in patients with hormone-receptor-positive (HR+) disease; however, no evidence of long-term toxicity was found (35). Regarding the AEs of neratinib on diarrhea, Cunningham et al. conducted a single-center retrospective study showing that neratinib was well tolerated by most patients as monotherapy or in combination with capecitabine when appropriate anti-diarrhea prophylaxis was given (36). Therefore, N+C should be considered as a useful alternative in third-line or later treatment of HER2+ mBC disease.

According to the result of one-way DSA, it is indisputable that most patients in China can bear the cost of N+C treatment. China’s antineoplastic drug market has been on a stable growth trend since health reform in 2009 (“2009 reform”). Overall, the drug price and national reimbursement negotiation provide an opportunity for timely inclusion of innovative anticancer medicines in the National Reimbursement Drug List (NRDL), which significantly improves the accessibility and affordability of anticancer medicines in China (37). Furthermore, a new centralized procurement, the national volume-based procurement (NVBP), was launched in November 2018 with the main goal of lowing drug prices and increasing the affordability of anticancer medicines (38). From the results of medical insurance negotiations announced by National Healthcare Security Administration (NHSA) in 2022, the average price reduction of drugs outside the medical insurance catalog was 62% and the average price reduction for antineoplastic drugs was 64.88%, including neratinib (39, 40). These initiatives have successfully overcome the problem that effective anticancer drugs fail to become the first choice for most patients and have provided an equal right to health care for everyone. For this reason, the research of pharmacoeconomic is of greater significance.

In this study, we added DoR as an analysis indicator to calculate the probability on the basis of commonly used OS and PFS, which was rare in previous pharmacoeconomic studies on BC. DoR refers to the time from the first evaluation of complete response (CR) or partial response (PR) to the first evaluation of PD or DE, which reflects the degree of long-term benefit of chemotherapy. As a result, this facilitates more appropriate decision making. In addition, we added subgroup analysis that gearing toward Asians, which demonstrated the applicability of our conclusions in Asia. To our knowledge, no other cost-effectiveness analysis had directly compared N+C and L+C in Asian subgroups. In China, patients, physicians, and policy makers may benefit from pharmacoeconomic studies regarding the Asian subgroups.

Our study has some limitations. First, the health utility values in this study were derived from a study of Chinese BC patients. Health utility values reflect social and cultural differences among populations in different countries, which might hinder the generalizability of our results. Second, whether the determination of transition probabilities reflects the true situation is unknown. Due to the lack of specialized clinical trials for pharmacoeconomic evaluation, this study was mainly based on the DEALE principal method, which calculated the transition probabilities from the literature data and assumed that the transition probabilities of the Markov model did not change during the study period. Third, the prices of chemotherapy drugs in our study were selected from the lowest global prices, which may affect the generalizability of the model across centers or countries since authentic clinical practice is always diverse. Finally, our analysis only focused on the costs with regard to health care; costs incurring outside the health care sector were failed to be included in our consideration.

Our analysis highlights the need for anticancer drug price to be reduced in the treatment of mBC. We hope that more effective anticancer drug could be evaluated by pharmacoeconomic and applied in the clinical practice to cure more patients.

## 5 Conclusion

In conclusion, our study confirmed that treatment with neratinib plus capecitabine was a cost-effective choice compared with lapatinib plus capecitabine in patients with HER2+ mBC who continues to progress during or after second-line HER2-targeted therapy. Therefore, neratinib plus capecitabine can become a third-line treatment option in China.

## Data availability statement

The original contributions presented in the study are included in the article/supplementary material. Further inquiries can be directed to the corresponding author.

## Ethics statement

All the data included in this analysis were derived from published literature and public data. No patient- identifiable data were applied or used. Therefore, institutional review board approval was not required.

## Author contributions

LR and QX contributed to the design of this study. NR collected the data. YZ and YY performed the analysis. LR prepared the manuscript. QX helped to revise the manuscript. All authors contributed to the article and approved the submitted version.
